# Editorial: Medical emergencies in psychiatry

**DOI:** 10.3389/fpsyt.2024.1458328

**Published:** 2024-07-24

**Authors:** Johannes M. Hennings, Ksenija Slankamenac

**Affiliations:** ^1^ Department of Psychiatry and Psychotherapy, Ludwig-Maximilians-University Hospital, Munich, Germany; ^2^ Department of Dialectical Behavioral Therapy, kbo Clinic Region Munich, Haar, Germany; ^3^ Emergency Department, University Hospital Zürich, Zurich, Switzerland

**Keywords:** medical emergency, psychiatry, emergency department, somatic symptom and related disorders, adverse (side) effects

Presentation of somatic symptoms in psychiatric patients are frequent for various reasons. Many psychiatric disorders increase the propensity to develop somatic disorders (1). At the same time, long-term psychopharmacological treatment harbors the risk to develop somatic side-effects, e.g., cardiac, or metabolic disturbances (2–4). Further, somatic symptoms are common accessory phenomena in many psychiatric conditions, like pain and weakness in mood disorders, cardiac and circulatory troubles in anxiety or addiction, impaired consciousness in post-traumatic stress disorder, bizarre bodily sensations in psychosis. A somatic symptomatology can even be the predominant presentation as in newly DSM-5 coined Somatic Symptom Disorder (SSD) that includes symptom complexes that have been classified previously as somatization disorder, hypochondriasis, pain disorder, and undifferentiated somatoform disorder (5).

As a pioneer project, the Research Topic “Medical Emergencies in Psychiatry” aims to provide a multi-professional interchange about somatic presentations in psychiatric patients.

First, mental disorders are associated with an increased propensity for somatic disorders, especially chronic physical conditions (1) such as diabetes (6) and metabolic syndrome (7). As shown in the case-control study of Gentil et al. of this issue, co-occurring chronic physical illness in patients with mental disorders increases the risk for emergency department (ED) use for psychiatric reasons in one year by 15%, the risk for psychiatric hospitalization by 63% compared to patients with mental disorders alone.

While Lithium, one of the most important and widely prescribed mood-stabilizing drug for bipolar and schizoaffective disorder, is known for its potential risk of serious adverse drug events (SAE) due to its narrow therapeutic index (8), the incidence of Lithium-related SAE is rather unknown. In their 17-year follow-up study that is part of the LiSIE (Lithium—Study into Effects and Side Effects) retrospective cohort study, Truedson et al. show that SAE related to treatment with Lithium for bipolar or schizoaffective disorder were uncommon but not rare. Especially older patients are at an elevated risk of Lithium-related SAE. The most common events were chronic Lithium intoxication, over-sedation, and cardiac/blood pressure-related events. Nevertheless, in their observation, SAE also occurred with other mood stabilizers and other psychotropic drugs.

Worldwide, the COVID-19 pandemic has been associated with an increased incidence and severity of pre-existent mental disorders and symptoms (9–11). Nevertheless, the effect of the COVID-19 pandemic on ED visits is a matter of ongoing debate since there is a shortage of research describing the evolution and nature of psychiatric ED visits. Aymerich et al. therefore thoroughly investigated 16,969 psychiatric ED visits before, during and after the COVID-19 pandemic. Unique to this study, they compared psychiatric ED visits in these different periods in one single general hospital increasing the comparability of their data. Although there was no rise of medical or traumatology visits, they found a disproportionate rise of up to 20% in the number of psychiatric consultations in the emergency department during the post-pandemic period, relative to both the pre-pandemic and pandemic periods, with a notable increase in consultations related to anxiety, suicidal ideation, and self-harm.

In severe cases, psychiatric disorders can lead to self-endangerment and a loss of decision-making abilities that can also affect the clinical management of physical illnesses. In those cases, forced medication can become necessary. Previous studies of forced medication investigated mainly emergency situations where forced medication is often managed with other coercive interventions such as seclusion but little is known about forced medication in non-emergencies (12). In a retrospective study, Meroni et al. analyzed forced medication in the Geneva University Hospital under non-emergency conditions but endangerment of others or oneself over a longer period. In line with previous studies, the diagnosis of a psychotic disorder was strongly associated with involuntary treatment but contrasting previous studies on emergency situations, women were at higher risk of being subjected to involuntary treatment because of self-endangerment in non-emergencies. Hence, the study of Meroni et al. describes an important subgroup of involuntary treated patients that appear to be distinct from patients involuntarily treated in emergency situations. Their data can thus help developing improved treatment conditions and minimizing harm to self-endangering patients.

Finally, Greenfield et al. presents three cases of functional neurological disorder (FND) in acute care settings. She highlights classical challenges when caring for these patients including over- or underutilization of diagnostic work-ups and consultation, over- or underutilization of psychopharmacological medications, and over- or undertreating a medical condition when a functional symptom is present. In each case, these lapses and errors caused the patient distress, additional treatments, care delays, delayed symptom remission as well as increased costs. The authors claim for an appropriate education and training, resources, and protocols in hospitals aiming to improve the identification and management of FND, and to prevent harm to patients.

Enjoy reading!

## Author contributions

JH: Conceptualization, Data curation, Formal analysis, Funding acquisition, Investigation, Methodology, Project administration, Resources, Software, Supervision, Validation, Visualization, Writing – original draft, Writing – review & editing. KS: Conceptualization, Data curation, Formal analysis, Funding acquisition, Investigation, Methodology, Project administration, Resources, Software, Supervision, Validation, Visualization, Writing – review & editing.
